# Active Living Collaboratives in the United States: Understanding Characteristics, Activities, and Achievement of Environmental and Policy Change

**DOI:** 10.5888/pcd10.120162

**Published:** 2013-02-07

**Authors:** Jill S. Litt, Hannah L. Reed, Rachel G. Tabak, Susan G. Zieff, Amy A. Eyler, Rodney Lyn, Karin Valentine Goins, Jeanette Gustat, Nancy O’Hara Tompkins

**Affiliations:** Author Affiliations: Hannah L. Reed, Colorado School of Public Health, Aurora, Colorado; Rachel G. Tabak, Amy A. Eyler, Washington University in St. Louis, St. Louis, Missouri; Susan G. Zieff, San Francisco State University, San Francisco, California; Rodney Lyn, Georgia State University, Atlanta, Georgia; Karin Valentine Goins, University of Massachusetts Medical School, Worcester, Massachusetts; Jeanette Gustat, Tulane University, New Orleans, Louisiana; Nancy O’Hara Tompkins, West Virginia University, Morgantown, West Virginia.

## Abstract

**Introduction:**

Changing the built environment to promote active lifestyles requires collaboration among diverse sectors. Multisectoral collaborative groups in the United States promote active lifestyles through environmental and policy changes. The objective of this study was to examine the characteristics of these collaborative groups and the extent to which they have achieved change.

**Methods:**

We identified, recruited, and interviewed the coordinators of active living collaborative groups in the United States. We used descriptive statistics to characterize groups by composition, stakeholder engagement, and the extent of environmental and policy change in 8 strategic areas.

**Results:**

Fifty-nine groups from 22 states participated in the study. Most groups had a diverse set of partners and used a range of activities to advance their agendas. Most groups achieved some form of environmental or policy change. On average, groups reported working on 5 strategy areas; parks and recreation (86%) and Safe Routes to School (85%) were named most frequently. More than half of groups reported their environmental initiatives as either in progress or completed. Groups reported the most success in changing policy for public plazas, street improvements, streetscaping, and parks, open space, and recreation. Complete Streets policy and zoning ordinances were the most frequently cited policy types. Engaging in media activities and the policy-making process in addition to engaging stakeholders appear to influence success in achieving change.

**Conclusion:**

Although many groups successfully worked on parks and recreation improvements, opportunities remain in other areas, including transit and infill and redevelopment. Additional time and resources may be critical to realizing these types of changes.

## Introduction

Physical inactivity is one of the great public health challenges of the 21st century (1–3). More than 80% of adults do not meet the recommended guidelines for both aerobic and muscle-strengthening activity (4). Individual attempts to initiate and sustain changes in physical activity behavior are generally unsuccessful in the absence of supportive physical and social environments (5). The built environment and related policies influence population-level physical activity (ie, movement that enhances health) and active living (ie, physical activity that results from daily routines) (6,7). Attributes of the built environment and policy directives can reduce barriers to physical activity, promote active lifestyles, and improve overall health, potentially reducing related health disparities (8,9).

Changing the built environment to promote active living requires cooperation among diverse sectors (10). The Centers for Disease Control and Prevention (CDC) has highlighted the importance of coordination among multiple sectors for improving the built environment (11). The Institute of Medicine has emphasized the importance of engaging nonhealth sectors in changing the built environment (12). Collaboration should involve people and organizations from multiple sectors (eg, planners, developers, media specialists, neighborhood residents, elected officials) and geographic strata (eg, state, regional, local, neighborhood) (11).

Collaborative groups that promote stakeholder engagement and interaction have been associated with increased relevance, feasibility, and long-term sustainability of initiatives (13). These groups have the potential to develop and maintain strategies to increase opportunities for physical activity by leveraging resources, sharing knowledge, and building relationships (12). They may also be effective in translating national guidelines and research recommendations into action by informing state and local policy-making processes, including agenda setting, policy formulation, and policy implementation (14,15).

Multisectoral collaborative groups in the United States are working to promote active lifestyles through environmental and policy changes. National, state, and local funding organizations have invested in initiating and maintaining these groups (7,9,16). The objective of this study was to examine the characteristics and activities of active living collaborative groups and the extent to which they have achieved environmental and policy changes.

## Methods

The cross-sectional study was designed by the Coalitions and Networks for Active Living (CANAL) research team, a subgroup of the CDC-funded Physical Activity Policy Research Network (PAPRN) (http://paprn.wustl.edu), and was conducted in 2011. The study was approved by the Colorado School of Public Health and Washington University in St. Louis institutional review boards.

### Recruitment and eligibility

We used the term “collaborative group” to represent coalitions, networks, partnerships, and alliances. We recruited representatives (“coordinators”) of active living collaboratives in the United States by using referrals from members of PAPRN and the National Society for Physical Activity Practitioners in Public Health (NSPAPPH) and approximately 50 alumni of the Physical Activity and Public Health Practitioners course. PAPRN represents approximately 17 organizations, and NSPAPPH, 90 organizations. We also searched the Internet to identify groups. Collaboratives were eligible to participate if they 1) focused on active living as a primary or secondary goal, 2) worked on policy and environmental change, 3) involved at least 3 partners from various sectors, and 4) existed for at least 1 year.

We identified 96 collaboratives and recruited 1 coordinator from each by e-mail. Of these, 59 (61%) collaboratives from 22 states participated. Six declined to participate, 3 cancelled, and 28 were invited but never responded. We made up to 3 attempts to contact each potential participant. The primary reason for refusal was lack of time.

### Data collection

We conducted structured telephone interviews from May through August 2011. We scheduled interviews and distributed the interview questions in advance to allow coordinators to review the questions and prepare their responses. Interviews lasted approximately 45 minutes and responses were entered into an electronic document. All participants answered all questions.

### Interview guide

The CANAL team developed the interview guide, using questions from previously validated instruments when possible. The guide included a mix of open- and closed-ended questions. We asked coordinators about their own organization and the collaborative.

We assessed leadership and management according to whether the collaborative had a designated lead agency and paid staff, the number of years the coordinator served in his or her position, whether the coordinator’s organization had expertise in public health or other areas (eg, land use, urban planning, transportation), the age of the collaborative, funding levels, and other items. We assessed the history of collaboration through 2 items adapted from the Wilder Collaboration Factors Inventory (17): 1) “Agencies in our collaborative have a history of working together” and 2) “Trying to solve problems through collaboration has been common in this community.” Response options to these 2 questions were 1 = strongly disagree, 2 = somewhat disagree, 3 = neutral, 4 = somewhat agree, and 5 = strongly agree. Responses were dichotomized to yes or no. We assessed composition of the collaborative by the number of partners and the range of sectors, disciplines, community perspectives, and areas of expertise represented. 

We asked coordinators about their agenda-setting activities: how they assessed their needs, created awareness through events, and engaged stakeholders in the policy-making process. To determine needs assessment, we asked coordinators about whether the following items had been completed or were in progress: 1) identification of a target population, 2) agreement on area to be served, 3) collection of information on opportunities for active living, and 4) formal meetings to decide on intervention strategy (18). For awareness activities, we asked whether the collaborative sponsored or supported walking/running events, safety events, awareness events, and other kinds of events. To measure engagement of stakeholders, we asked about 10 activities on a 5-point Likert scale (1 = never, 2 = rarely [<2 times], 3 = sometimes [2–5 times], 4 = often [most of the time], 5 = very frequently or ongoing) (19). 

Coordinators were asked whether their collaborative had used any of the following approaches to promote active living or physical activity in the community: environmental change (eg, trail building), policy change (eg, complete streets), program development (bike/walk-to-work event), educational programs (eg, bike safety), social media (eg, Facebook), or social marketing (eg, advertisements). We asked whether the collaborative had identified any of the following areas as a core strategy: parks, open spaces, and recreation facilities; transit and parking; children’s play areas; public plazas (ie, community destinations, such as gardens and farmers’ markets); streetscaping (eg, traffic calming); street improvements (eg, street connectivity); infill and redevelopment (eg, mixed use development); and Safe Routes to School. These strategy areas are consistent with the New York City Active Design Guidelines (20) and themes developed by LiveWell Colorado (www.livewellcolorado.org) and Kaiser Permanente (http://info.kaiserpermanente.org/communitybenefit/html/our_work/global/our_work_3.html). We asked about the extent to which the collaborative had addressed the strategy; respondents replied according to the following scale: 1 = improvements have been discussed, 2 = improvements have been planned, 3 = improvements have been funded, 4 = improvements are in progress, and 5 = improvements have been completed. We asked whether policies were in place to address the strategy and whether any improvement had required a change in policy or development of new policy. For policies in place or new policy, we asked what type of policy was required. Finally, if a change in policy was required by the improvement, we asked about the status of the new policy. Possible responses were 1 = policy gap identified but no further action, 2 = policy gap identified and discussion initiated, 3 = policy drafted, 4 = policy adopted or approved, and 5 = cannot be determined.

To ensure content validity, interview questions were reviewed by academic partners, planners, and public health practitioners and pretested with 4 nonenrolled groups before the study began. We made only minor changes after the pretest.

### Data analyses

We entered interview data directly into Microsoft Access 2007 (Microsoft Corporation, Redmond, Washington). We tabulated the data and summarized the distribution, central tendency, and dispersion by using SAS version 9.2 (SAS Institute, Cary, North Carolina). To augment data provided by interviews, we assessed the mean poverty level of the areas served by the collaborative by the percentage of families in poverty (21) and identified levels of physical inactivity by state (22).

We assigned a score to each group for each strategy area on the basis of the status of its environmental improvements and related policy change. We averaged the scores for all strategy areas on which a group worked. We dichotomized groups according to their scores into high and low levels of policy change (high = policies have been drafted, adopted or approved; low = other responses) and high and low levels of environmental change (high = improvements are in progress or have been completed; low = other responses). We then created 4 groups: high levels of both policy and environmental change (High–High), high level of policy change and low level of environmental change (High–Low), low level of policy change and high level of environmental change (Low–High), and low levels of both policy and environmental change (Low–Low). We used the Fisher exact test and analysis of variance to test for differences between groups. 

## Results

**Figure 1:**
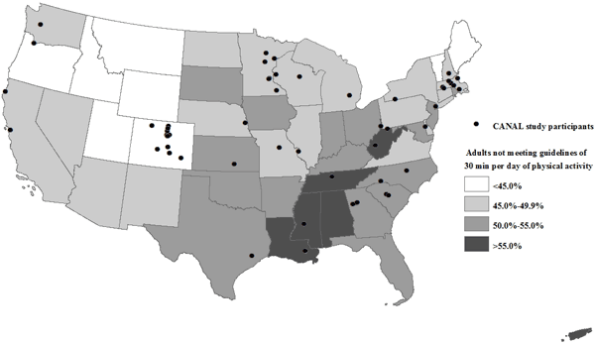
Location of coordinators who participated in the Coalitions and Networks for Active Living (CANAL) Study, by state estimates of physical inactivity among adults. Source for data on physical inactivity: Centers for Disease Control and Prevention (22). **Table:** 

Active living groups were located throughout the United States in states that varied by levels of physical inactivity (Figure 1). Most (78%) groups worked locally; the rest addressed state-wide issues. Coordinators represented government organizations (41%) and nonprofit organizations (59%) (Table 1). Twenty-five percent of the groups served urban areas, whereas 9% served suburban areas, 17% served rural areas, and 49% served mixed areas. They served areas of various geographic sizes: neighborhoods (6.5% of collaboratives), city/towns (36%), counties (29%), regions (6.5%), and states (22%). The percentage of families living in poverty was consistent for collaboratives serving city/towns (13%), counties (9%), regions (7%), and states (11%) but was higher for groups serving neighborhoods (26%). 

State	% of Adults Not Meeting Guidelines of 30 Minutes/Day Physical Activity	No. of Collaboratives
Colorado	<45.0%	12
Massachusetts	45.0%–49.9%	7
Minnesota	45.0%–49.9%	6
Louisiana	>55.0%	5
West Virginia	>55.0%	3
Georgia	50.0%–55.0%	3
California	45.0%–49.9%	3
South Carolina	50.0%–55.0%	2
Missouri	45.0%–49.9%	2
North Carolina	50.0%–55.0%	2
Wisconsin	45.0%–49.9%	2
New York	45.0%–49.9%	2
Mississippi	>55.0%	1
Tennessee	>55.0%	1
Michigan	45.0%–49.9%	1
Texas	50.0%–55.0%	1
Kansas	50.0%–55.0%	1
Delaware	45.0%–49.9%	1
Nebraska	45.0%–49.9%	1
Washington	45.0%–49.9%	1
New Hampshire	45.0%–49.9%	1
Oregon	<45.0%	1

### Collaborative leadership and management

Most coordinators (76%) reported that their collaborative had designated a lead agency. These lead agencies were housed in health departments (35%), nonprofit organizations (15%), and health care agencies (9%). Only 33% of the coordinators were in their position for 3 or more years (Table 1). Seventy percent of coordinators reported their collaborative had expertise in public health, but only 37% reported expertise in land use or urban planning, and only 32% reported expertise in transportation.

**Table 1:** Characteristics of Collaborative Groups (N = 59) Participating in the Coalitions and Networks for Active Living (CANAL) Study, 2011

Characteristic	No. (%)
**Sector affiliation of organization represented by coordinator^a^**	
Nonprofit	35 (59)
Government	24 (41)
Private	3 (5)
**Coordinator’s no. of years in position**	
<Half	4 (7)
Half to 1	13 (22)
>1 to 3	23 (39)
>3 to 5	11 (19)
>5	8 (14)
**Age of collaborative group, y**	
1–3	14 (24)
4–6	26 (44)
≥7	19 (32)
**Paid staff**	
Yes	50 (85)
No	9 (15)
**No. of active partners**	
1–10	12 (20)
11–30	26 (44)
31–50	12 (20)
>50	9 (16)
**Funding, $**	
<25,000	7 (12)
25,000–99,999	14 (24)
100,000–199,999	15 (25)
200,000–499,999	11 (19)
≥500,000	6 (10)
Don’t know or none	6 (10)

a Survey respondents could choose more than 1 category.

Almost half (44%) of collaboratives had worked with their partners for 4 to 6 years; 83% had a history of working together, and 76% solved problems collaboratively. Most (85%) collaboratives had paid staff. Most established written goals or objectives (97%), wrote a mission or a vision statement (93%), prepared a strategic plan or annual report (85%), and used a theoretical framework or conceptual model to guide their priorities and actions (73%).

### Collaborative composition

Most collaborative groups had a diverse membership that represented a range of sectors (public, government, private), disciplines (public health, planning, agriculture, sports and fitness), and perspectives (residents, local leaders, universities, schools, business leaders, and faith-based organizations). Forty-four percent of collaboratives reported having 11 to 30 active partners (Table 1). Most had expertise in public and/or environmental health (95%), fitness and sport (83%), parks and recreation (83%), land use planning (80%), and transportation (76%). Only half had media-related expertise (51%), and a smaller percentage had expertise in law enforcement/safety (46%), housing (32%), and mental health (29%).

### Agenda-setting activities

Most coordinators reported completing assessment activities and supporting community events (Table 2). Two-thirds of coordinators reported that community leaders often or very frequently participated or endorsed a collaborative-sponsored event. Most (88%) collaboratives served on active living advisory councils, 75% engaged with elected officials or staff, 66% partnered with planning and design experts, and 63% engaged school district or board officials often or very frequently. Fewer collaboratives often or very frequently engaged in such activities as media communication or advocacy (52%), written advocacy (eg, press release, policy analysis, report with policy recommendations) (41%), partnering with elected officials to author policy documents (36%), or offering testimony in policy, legal, or judicial hearings (21%).

**Table 2:** Agenda-Setting Activities: Needs Assessment, Community Events, and Engagement of Stakeholders in Policy-Making Processes, the Coalitions and Networks for Active Living (CANAL) Study, 2011

Activity	No. (%) of Groups (N = 59)
**Needs Assessment**	
**Identified a target population**	
Not completed or in progress	10 (17)
Completed	49 (83)
**Agreed on area to be served**	
Not completed or in progress	7 (12)
Completed	52 (88)
**Collected information on opportunities for physical activity**	
Not completed or in progress	18 (30)
Completed	41 (70)
**Held formal meeting to decide on intervention strategy**	
Not completed or in progress	10 (17)
Completed	49 (83)
**Community Events Sponsored or Supported^a^**	
Walking/running	39 (66)
Bicycling	36 (61)
Safety	36 (31)
Street opening	14 (24)
Awareness events	47 (80)
Networking events	43 (73)
**Stakeholder Activities**	
**Engages with elected officials or staff**	
Sometimes/rarely (<5 times)	15 (25)
Often (most of the time)	15 (25)
Very frequently (or ongoing)	29 (50)
**Leaders participate/endorse a collaborative sponsored event**	
Sometimes/rarely/never (<5 times)	20 (34)
Often (most of the time)	21 (36)
Very frequently (or ongoing)	18 (30)
**Engages with school district/board officials**	
Sometimes/rarely (<5 times)	22 (37)
Often (most of the time)	15 (26)
Very frequently (or ongoing)	22 (37)
**Partners with elected official to author policy document**	
Rarely/never (<2 times)	16 (27)
Sometimes (2–5 times)	22 (37)
Very frequently/often	21 (36)
**Engages in written advocacy**	
Rarely/never (<2 times)	13 (22)
Sometimes (2–5 times)	22 (37)
Often (most of the time)	19 (32)
Very frequently (or ongoing)	5 (9)
**Offers testimony in policy, legal, or judicial hearing**	
Never	12 (20)
Rarely (<2 times)	12 (20)
Sometimes (2–5 times)	22 (37)
Often (most of the time)	8 (14)
Very frequently (or ongoing)	4 (7)
Does not know	1 (2)
**Engages in media communication/advocacy**	
Rarely/never (<2 times)	8 (14)
Sometimes (2–5 times)	20 (34)
Often (most of the time)	19 (32)
Very frequently (or ongoing)	12 (20)
**Serves on physical activity or active living workgroup/advisory council**	
Sometimes/rarely/never (<5 times)	7 (12)
Often (most of the time)	18 (30)
Very frequently (or ongoing)	34 (58)
**Recruits new partners with policy expertise**	
Rarely/never (<2 times)	9 (15)
Sometimes (2–5 times)	22 (37)
Often (most of the time)	21 (36)
Very frequently (or ongoing)	7 (12)
**Partners with planning/design practitioners**	
Rarely/never (<2 times)	6 (10)
Sometimes (2–5 times)	14 (24)
Often (most of the time)	13 (22)
Very frequently (or ongoing)	26 (44)

a Survey respondents could choose more than 1 category.

### Environmental and policy changes

**Figure 2:**
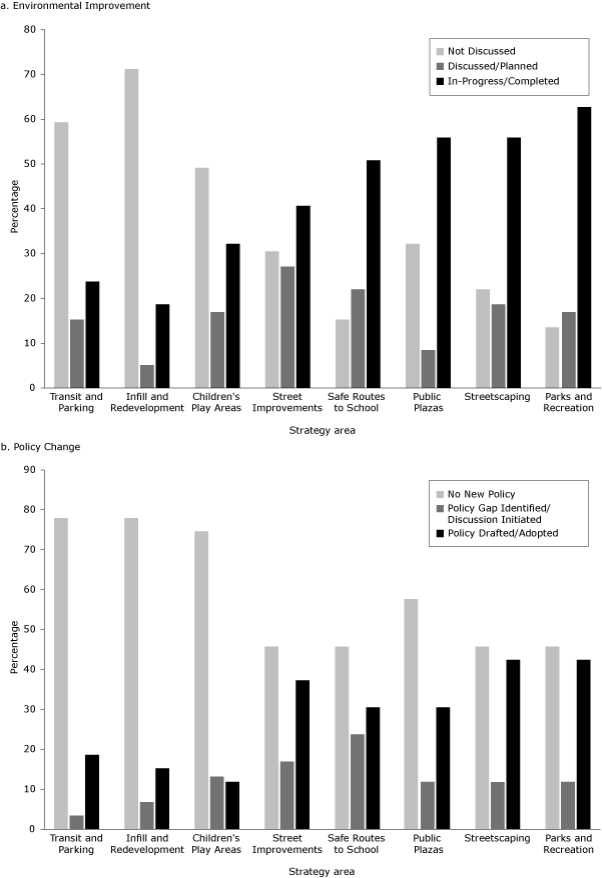
Environmental and policy change in 8 core strategy areas among 59 collaborative groups, the Coalitions and Networks for Active Living (CANAL) Study, 2011. **Table:** 

Nearly all collaboratives used environmental (90%) or policy (93%) approaches to promote active living. They also used program development (83%), education (78%), social media (69%), and social marketing (56%). Collaboratives worked on an average of 5 core strategy areas; 86% identified parks and recreation; 85%, Safe Routes to School; 78%, street improvements; and 69%, streetscaping. Fewer groups reported transit and parking-related projects (41%) and infill and redevelopment-related projects (29%). In 4 strategy areas, more than half of groups reported their strategy as either in progress or completed (Figure 2).

**a. Environmental Improvement**			

**Strategy area**	**Not Discussed, %**	**Discussed/Planned, %**	**In-Progress/Completed, %**

Transit and parking	59	15	24
Infill and redevelopment	71	5	19
Children’s play areas	49	17	32
Street improvements	31	27	41
Safe Routes to School	15	22	51
Public plazas	32	8	56
Streetscaping	22	19	56
Parks and recreation	14	17	63

**b. Policy Change**			

**Strategy area**	**No New Policy, %**	**Policy Gap Identified/Discussion Initiated, %**	**Policy Drafted/Adopted, %**

Transit and parking	78	3	19
Infill and redevelopment	78	7	15
Children’s play areas	75	13	12
Street improvements	46	17	37
Safe Routes to School	46	24	31
Public plazas	58	12	31
Streetscaping	46	12	42
Parks and recreation	46	12	42

Most groups cited existing policy for each strategy area on which they were working; 93% reported changes to existing policy or the development of new policy in at least 1 strategy area. Complete Streets policy and zoning ordinances were the most frequently cited policy types. Comprehensive plans, other plan types (eg, parks, open space, transportation, bike/pedestrian), joint-use agreements, and organizational policies were also commonly cited by groups.

Compositional and community engagement characteristics varied by level of environmental and policy change (Table 3). Collaboratives whose environmental improvements were in progress or completed and whose policies were drafted or adopted had been together longer and used more types of community events to engage stakeholders. Groups reporting more progress in environmental and policy change more frequently engaged in political and policy processes. They also reported using social marketing and social media to support their work.

**Table 3:** Average Characteristics of Collaboratives (N = 59) in 4 Levels of Policy and Environmental Change

Characteristic	High Level of Policy Change–High Level of Environment Change^a^ (n = 10)	High Level of Policy Change–Low Level of Environment Change^b^ (n = 15)	Low Level of Policy Change–High Level of Environment Change^c^ (n = 14)	Low Level of Policy Change–Low Level of Environment Change^d^ (n = 20)	*P* Value
Age of group, y	7.8	6.3	5.4	4.9	.15^e^
Annual funding, $	233,850	260,430	230,821	102,310	.44^e^
Has a designated lead agency, %	70	73	71	85	.70^f^
No. of event types hosted (of 6 possible event types)	5	4	5	3	.34^f^
Frequency of media engagement	Very frequently	Often	Sometimes	Sometimes	.02^f^
Frequency of testimony	Sometimes	Sometimes	Rarely	Sometimes	.03^f^
Frequency of partnering with planning/design practitioners	Very frequently	Very frequently	Very frequently	Sometimes	.10^f^
Used social marketing, %	80	67	57	35	.09^f^
Used social media, %	90	73	86	40	.01^f^

a Policies have been drafted and adopted or approved, and environmental improvements are in progress or completed.

b Policies are drafted and adopted or approved, and environmental improvements have been discussed, planned, or funded only.

c There are no new policies, a policy gap has been identified but no further action has been taken, or a gap has been identified and discussion has been initiated, and environmental improvements are in progress or completed.

d There are no new policies, a policy gap has been identified but no further action has been taken, or a gap has been identified and discussion has been initiated, and environmental improvements have been discussed, planned, or funded only.

e Determined by analysis of variance.

f Determined by Fisher exact test.

## Discussion

To our knowledge, this study is the first to explore collaboratives working to improve active living environments and highlight the range of activities used by practitioners to facilitate change. Public health and planning guidelines suggest that active living interventions are most effective when a combination of environmental approaches from various domains (ie, intrapersonal, social, physical environment, and policy) is used (6,23). In general, groups appear to be advancing an environmental- and policy-change agenda that is consistent with international and national guidelines and recommendations (12,24,25).

Most groups in this study involve partners from multiple sectors. Such involvement is important to achieving environmental and policy change (15,26). Expansion of partners beyond the health sector marks a shift toward more strategic inclusion of needed expertise (27,28). Strategic involvement of key partners plays a crucial role in informing, designing, and promoting a policy agenda (29). Broader participation can expand the knowledge, skills, and resources necessary to navigate complex processes that span multiple sectors and levels of government and reach communities in various settings (eg, work, home, school) (11,30).

Building relationships with community members and decision makers is another element for collaborative groups to facilitate environmental change. Collaboratives use different tactics and activities in varying degrees to advance their agendas. These tactics and activities serve multiple purposes: 1) elicit local knowledge to inform the design and implementation of strategies; 2) motivate core partners and the broader community of practitioners and citizens to get involved; 3) raise awareness about the problems being addressed and the need for change at multiple levels; and 4) align group-specific political and policy priorities (19,30,31). Needs assessments are unique in their ability to engage partners, policy makers, elected officials, media, and community members in agenda-setting and planning processes and create community awareness and buy-in (32,33). Although groups appear to be building relationships with elected and appointed officials, they vary in their use of other advocacy activities such as media communication and written testimony. This variability may reflect different levels of coordinator and partner expertise in advocacy and perceptions by collaborative coordinators that they are not permitted to engage in advocacy because of funding or other restrictions (34). Finally, groups using social media and marketing tools may be at an advantage for advancing their active living agendas. Such tools can serve to create awareness, increase public input, and build community support for active living planning processes.

Partner diversification, resource leverage, broad community involvement, and engagement in the political process set the stage for the adoption and implementation of environmental and policy change. The work of groups to identify the issues and raise community awareness also builds public and political will for an active living policy agenda. By hosting events such as open street initiatives or bike-to-work days, groups are able to raise awareness among a diverse group of stakeholders and foster a sense of urgency about the built environment and active lifestyles (23,33). Additionally, these groups extend the visibility of their events by soliciting endorsements from elected officials and community leaders and by investing in social marketing and media campaigns. Groups use these opportunities to showcase their work to key decision makers as a gateway to promoting their policy agendas (19). Our study shows that engaging citizens through community meetings, building political support by working with elected officials and staff (eg, city council, mayor, county commissioner), and working with designers (eg, private consultants, public works, engineering, transit authority) are being used to negotiate the policy-making process.

Our findings illustrate the range and related success of environmental and policy approaches used by groups to promote active living. Although groups are universally working on built environment improvements, these projects represent smaller-scale improvements such as community gardens, public plaza improvements, and park enhancements, for example. These smaller-scale successes may reflect gateway strategies that are well defined, evidence-based, affordable, and achievable within shorter time frames. Only a small percentage of groups report actions in the areas of transit, parking, and infill redevelopment. These strategy areas require larger-scale investments, strategic relationships with agencies and people who may be external to the collaborative (35), and time for developing related policies (33).

This study has several limitations. First, our cross-sectional design limited our ability to explore the temporal aspects of the group agenda-setting processes. Second, the sample was constructed through a nomination process, so results may reflect the work of groups that have greater visibility and more successful initiatives and may not be generalizable to all active living collaboratives. Third, collection of data from group coordinators may bias results in a positive direction because of the professional and personal investment of coordinators.

This study describes the work accomplished by collaborative groups, including community organizations, local government agencies, and the private sector, to promote active living in the United States. These findings provide insights into the composition and activities of these groups and their environmental and policy approaches. Achieving environmental and policy change requires time, social and political connections, and financial resources. For funders and government entities investing in community-level environmental and policy change efforts, such knowledge will be useful for setting future priorities and developing expectations, training, and technical assistance to plan, coordinate, implement, and sustain collaborative-led strategies.

## References

[1] Blair SN , Kohl HW 3rd , Barlow CE , Paffenbarger RS , Gibbons LW , Macera CA . Changes in physical fitness and all-cause mortality: a prospective study of healthy and unhealthy men. JAMA 1995;273(14):1093–8. 10.1001/jama.1995.03520380029031 7707596

[2] Blair SN . Physical inactivity: the biggest public health problem of the 21st century. Br J Sports Med 2009;43(1):1–2. 19136507

[3] Pratt M , Epping JN , Dietz WH . Putting physical activity into public health: a historical perspective from the Centers for Disease Control and Prevention. Prev Med 2009;49(4):301–2. 10.1016/j.ypmed.2009.06.011 19555709

[4] US Department of Health and Human Services. 2008 physical activity guidelines for Americans. Washington (DC): US Department of Health and Human Services; 2008.

[5] Alvaro C , Jackson LA , Kirk S , McHugh TL , Hughes J , Chircop A , Moving governmental policies beyond a focus on individual lifestyle: some insights from complexity and critical theories. Health Promot Int 2011;26(1):91–9. 10.1093/heapro/daq052 20709791 PMC3033735

[6] Sallis JF , Bauman A , Pratt M . Environmental and policy interventions to promote physical activity. Am J Prev Med 1998;15(4):379-97. 9838979 10.1016/s0749-3797(98)00076-2

[7] Schmid TL , Pratt M , Witmer L . A framework for physical activity policy research. J Phys Act Health 2006;3(Suppl 1):S20–9. 28834511 10.1123/jpah.3.s1.s20

[8] Brownson RC , Haire-Joshu D , Luke DA . Shaping the context of health: a review of environmental and policy approaches in the prevention of chronic diseases. Annu Rev Public Health 2006;27:341–70. 10.1146/annurev.publhealth.27.021405.102137 16533121

[9] Frank L , Kavage S . A national plan for physical activity: the enabling role of the built environment. J Phys Act Health 2009;6(Suppl 2):S186–95. 20120128

[10] Hutch DJ , Bouye KE , Skillen E , Lee C , Whitehead L , Rashid JR . Potential strategies to eliminate built environment disparities for disadvantaged and vulnerable communities. Am J Public Health 2011;101(4):587–95. 10.2105/AJPH.2009.173872 21389288 PMC3052324

[11] Varda DM , Chandra A , Stern SA , Lurie N . Core dimensions of connectivity in public health collaboratives. J Public Health Manag Pract 2008;14(5):E1–7. 18708879 10.1097/01.PHH.0000333889.60517.46

[12] Institute of Medicine Committee on Accelerating Progress in Obesity Prevention Food and Nutrition Board. Accelerating progress in obesity prevention: solving the weight of the nation. Glickman D, Parker L, Sim L, Del Valle Cook H, Miller E, editors. Washington (DC): National Academies Press; 2012. 24830053

[13] Naylor PJ , Macdonald HM , Reed KE , McKay HA . Action Schools! BC: a socio-ecological approach to modifying chronic disease risk factors in elementary school children. Prev Chronic Dis 2006;3(2). 16539801 PMC1563946

[14] Roussos ST , Fawcett SB . A review of collaborative partnerships as a strategy for improving community health. Annu Rev Public Health 2000;21:369–402. 10.1146/annurev.publhealth.21.1.369 10884958

[15] Mays GP , Scutchfield FD . Improving public health system performance through multiorganizational partnerships. Prev Chronic Dis 2010;7(6):A116. 20950523 PMC2995603

[16] Schilling JM , Giles-Corti B , Sallis JF . Connecting active living research and public policy: transdisciplinary research and policy interventions to increase physical activity. J Public Health Policy 2009;30(Suppl 1):S1–15. 10.1057/jphp.2008.59 19190567

[17] Mattessich P , Monsey B . Community building: what makes it work? A review of factors influencing successful community building. St. Paul (MN): Amherst Wilder Foundation; 1997.

[18] Jasuja GK , Chou CP , Bernstein K , Wang E , McClure M , Pentz MA . Using structural characteristics of community coalitions to predict progress in adopting evidence-based prevention programs. Eval Program Plann 2005;28:173–84. 10.1016/j.evalprogplan.2005.01.002

[19] Minkler M , Garcia A , Williams J , LoPresti T , Lilly J . Si se puede: using participatory research to promote environmental justice in a Latino community in San Diego, California. J Urban Health 2010;87(5):796–812. 10.1007/s11524-010-9490-0 20683782 PMC2937121

[20] New York City Department of Design and Construction. Active design guidelines: promoting physical activity and health in design. http://www.nyc.gov/adg. Accessed January 4, 2013.

[21] American community survey 2006–2010 (5-year estimates). New York (NY): Social Explorer; 2012. www.socialexplorer.com. Accessed January 2, 2013.

[22] Centers for Disease Control and Prevention. Behavioral risk factor surveillance system survey data. Atlanta (GA): US Department of Health and Human Services; 2008. http://www.cdc.gov/brfss/. Accessed February 13, 2012.

[23] Sallis JF , Cervero RB , Ascher W , Henderson KA , Kraft MK , Kerr J . An ecological approach to creating more physically active communities. Annu Rev Public Health 2006;27:297–322. 10.1146/annurev.publhealth.27.021405.102100 16533119

[24] Edwards P , Tsouros A . Promoting physical activity and active living in urban environments: the role of local governments. Copenhagen (DK): WHO Regional Office for Europe; 2006.

[25] Centers for Disease Control and Prevention. The CDC guide to strategies to increase physical activity in the community. Atlanta (GA): US Department of Health and Human Services; 2011.

[26] Fawcett S , Schultz J , Watson-Thompson J , Fox M , Bermby R . Building multisectoral partnerships for population health and equity. Prev Chronic Dis 2010;7(6):A118. 20950525 PMC2995607

[27] Varda D , Shoup JA , Miller S . A systematic review of collaboration and network research in the public affairs literature: implications for public health practice and research. Am J Public Health 2012;102(3):564–71. 10.2105/AJPH.2011.300286 22021311 PMC3487657

[28] Khan LK , Sobush K , Keener D , Goodman K , Lowry A , Kakietek J , Recommended community strategies and measurements to prevent obesity in the United States. MMWR Recomm Rep 2009;58(RR-7):1–26. 19629029

[29] Korfmacher KS . Boundary networks and Rochester’s “smart” lead law: the use of multidisciplinary information in a collaborative policy process. New Solut 2010;20(3):317–36. 10.2190/NS.20.3.f 20943475 PMC3779540

[30] Lasker RD , Weiss ES . Broadening participation in community problem solving: a multidisciplinary model to support collaborative practice and research. J Urban Health 2003;80(1):14–47; discussion 48–60. 10.1093/jurban/jtg014 12612096 PMC3456118

[31] Padget SM , Bekemeier B , Berkowitz B . Collaborative partnerships at the state level: promoting systems changes in public health infrastructure. J Public Health Manag Pract 2004;10(3):251–7. 15253521 10.1097/00124784-200405000-00009

[32] Green CG , Klein EG . Promoting active transportation as a partnership between urban planning and public health: the Columbus Healthy Places program. Public Health Rep 2011;126(Supp 1):41–9. 21563711 10.1177/00333549111260S107 PMC3072902

[33] Emery J , Crump C , Hawkins M . Formative evaluation of AARP’s Active for Life campaign to improve walking and bicycling environments in two cities. Health Promot Pract 2007;8(4):403–14. 10.1177/1524839906292179 17494950

[34] Dorfman L , Gonzalez P . Media advocacy: a strategy for helping communities change policy. In: Minkler M, editor. Community organizing and community building for health and welfare. Third edition. New Brunswick (NJ): Rutgers University Press; 2012. p. 407–20.

[35] Allen NE , Javdani S , Lehrner AL , Walden AL . “Changing the text”: modeling council capacity to produce institutionalized change. Am J Community Psychol 2012;49(3–4):317–31. 10.1007/s10464-011-9460-z 21842302

